# Interactions Between Intestinal Microbiota and Neural Mitochondria: A New Perspective on Communicating Pathway From Gut to Brain

**DOI:** 10.3389/fmicb.2022.798917

**Published:** 2022-02-24

**Authors:** Yao Zhu, Ying Li, Qiang Zhang, Yuanjian Song, Liang Wang, Zuobin Zhu

**Affiliations:** ^1^Xuzhou Engineering Research Center of Medical Genetics and Transformation, Key Laboratory of Genetic Foundation and Clinical Application, Department of Genetics, Xuzhou Medical University, Xuzhou, China; ^2^Medical Technology College, Xuzhou Medical University, Xuzhou, China; ^3^Department of Bioinformatics, School of Medical Informatics and Engineering, Xuzhou Medical University, Xuzhou, China; ^4^Jiangsu Key Laboratory of New Drug Research and Clinical Pharmacy, School of Pharmacy, Xuzhou Medical University, Xuzhou, China

**Keywords:** intestinal microbiome, mitochondria, microbiota-gut-brain axis, brain, gut

## Abstract

Many studies shown that neurological diseases are associated with neural mitochondrial dysfunctions and microbiome composition alterations. Since mitochondria emerged from bacterial ancestors during endosymbiosis, mitochondria, and bacteria had analogous genomic characteristics, similar bioactive compounds and comparable energy metabolism pathways. Therefore, it is necessary to rationalize the interactions of intestinal microbiota with neural mitochondria. Recent studies have identified neural mitochondrial dysfunction as a critical pathogenic factor for the onset and progress of multiple neurological disorders, in which the non-negligible role of altered gut flora composition was increasingly noticed. Here, we proposed a new perspective of intestinal microbiota – neural mitochondria interaction as a communicating channel from gut to brain, which could help to extend the vision of gut-brain axis regulation and provide additional research directions on treatment and prevention of responsive neurological disorders.

## Introduction

**FIGURE 1:**
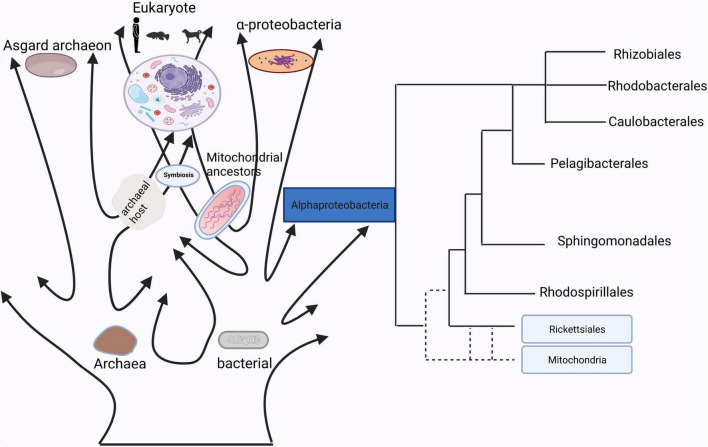
The homology between mitochondria and bacteria.

Human body is a super organism composed of own cells and resident microorganisms. In the long-term co-evolutionary process, human gut microbes, and the hosts constantly selected and adapted to each other, bringing about a close symbiotic relationship presently (Moeller et al., 2016). While microbiota exist in many body sites such as oral cavity, vagina, airways, and skin, etc., we focused only on the gut microbiota in this study as its interplay with systemic health is the most extensively documented (van de Guchte et al., 2018). In consideration of its distributive peculiarity, the gut microbiota was primarily proposed to have specific interactions with the host digestive system, which served as the main study topic for past decades. In recent years, a mass of research has identified that gut microbiota and corresponding bacterial metabolites can target the brain through various pathways, such as nervous conduction (enteric nerve, vagus nerve, etc.) (Fulling et al., 2019), hypothalamic–pituitary–adrenal (HPA) axis (McNeilly et al., 2010), and enteric endocrine and immune response (Fung, 2020; Morais et al., 2020), etc., (Figure 1). However, the specific regulatory mechanisms in these channels remain largely unclear.

Brain is one of the most energy-consuming organ in the body (Karbowski, 2007). Neural mitochondria can not only provide energy for maintaining brain homeostasis, but are also central regulators of cognitive function as well as fate determinant for neural stem cells (Khacho et al., 2016; Iwata et al., 2020). It has been widely reported that mitochondrial dysfunction can accelerate senescence of neural cells and facilitate the onset of multiple neurological diseases (Nguyen et al., 2014). In parallelly, a large amount of evidence confirmed that gut microbiota composition played critical roles in regulating the physiological and pathological functions of the brain. Therefore, considering the common ancestries, similar mechanisms, similar goals, and similar structures between gut microbiota and mitochondria (Franco-Obregon and Gilbert, 2017), is it possible that neural mitochondria are direct targets of intestinal microflora and function as key mediators regulating gut-brain interaction?

## Mitochondria Have a Close Relationship With Bacteria

In Sagan (1967), first proposed the hypothesis that mitochondria evolved from bacteria. Currently, determining the nature of the bacterial lineages that gave rise to mitochondrial ancestors is still a hotly debated topic (Figure 1). Although many studies have already showed that mitochondrion originated from within the bacterial phylum *Alphaproteobacteria*, the phylogenetic relationship of the mitochondrial endosymbiont to extant *Alphaproteobacteria* is yet unclear (Fan et al., 2020), while other studies support the idea that mitochondria evolved from an ancestor related to *Rickettsiales* (Andersson et al., 1998; Wang and Wu, 2015). It is true that mitochondria have a bacterial origin and do share many proteins that mediate similar or even the same metabolic processes (Karlberg et al., 2000). Besides that, the use of antibiotics such as quinolones, aminoglycosides and poplar polysaccharide antibiotics can induce mitochondria dysfunction due to similarities in their structures with bacteria (Kalghatgi et al., 2013; Lleonart et al., 2017). For instance, that quinolones target mtDNA topoisomerases (Gootz et al., 1990) and bacterial gyrases (Wolfson and Hooper, 1985), aminoglycosides target both mitochondrial (Hutchin and Cortopassi, 1994) and bacterial ribosomes (Davis, 1987). Reversibly, mitochondrial-targeted antioxidants could also function as effective antibiotics (Nazarov et al., 2017). These studies suggested that mitochondria have a close relationship with bacteria, which indicates a possibility of information exchange between gut microbiota and mitochondria.

## Intestinal Microbiota Directly Regulate Brain Function Through Intestinal Epithelium and Gastrointestinal Nerve

**FIGURE 2:**
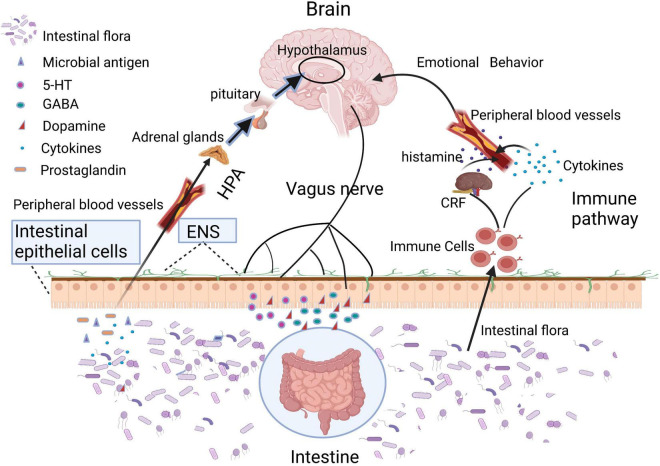
Intestinal microbiota or its metabolites can directly regulate brain function through HPA axis, vagus nerve, and immune pathway.

Intestinal epithelium and gastrointestinal nerves are the first sites of interactions between microbes and hosts (Figure 2). Moreover, many toxins produced by gut microbiota can lead to mitochondrial dysfunctions. For example, when the host was infected by pathogenic bacteria, mitochondria will be activated by lipopolysaccharides and other toxins released by the gut microbiota, inducing the accumulation of mitochondrial reactive oxygen species (ROS), which sequentially mediate intestinal inflammation (Mills et al., 2016). In addition, toxins secreted by certain species of *Clostridium* could inhibit the mitochondrial ATP-sensitive potassium channels, leading to mitochondrial membrane hyperpolarization, cell apoptosis, and intestinal epithelial barrier disruption (Matarrese et al., 2007; Berger et al., 2016; Crakes et al., 2019). The increased intestinal permeability enabled the translocation of damaging substances or pathogens into the intestinal epithelium and gastrointestinal nerves. Vagus nerve, an important link in the gut-brain axis, is able to sense microbial metabolites through its afferents, which transfers gut information to the central nervous system (Bonaz et al., 2018; Yu et al., 2020). Moreover, mitochondria are an important source of damaged cells release endogenous messengers (DAMPs), the release of these mitochondrial ROS have role of signaling in neuroinflammation and neurodegenerative diseases (Hsieh and Yang, 2013). DAMPs can also activate the innate immune system (Taanman, 1999), while innate immunity further reacts to different insults that may challenge the integrity of the central nervous system (CNS; Liu et al., 2014).

## Intestinal Microbiota Remote Control the Physiological and Pathological Functions of the Brain Through the Mitochondrial Pathway

Experimental evidence has been showing that metabolites produced by gut microbes interact directly with brain mitochondria, providing new insights into the communication pathway from gut to brain. In specificity, intestinal infection caused by lipopolysaccharides from gram-negative bacteria could trigger mitochondrial antigen presentation (MitAP) in autoimmune CD8 + T cells, which could enter the brain to attack dopamine neurons and cause a sharp decrement in the density of dopaminergic axon expansion in the striatum, leading to movement disorders like Parkinson’s disease (PD) in mice (Matheoud et al., 2019). Effects of pathogenic bacteria on mitochondria can also include morphological and functional changes. Listeriolysin O (LLO) secreted by *Listeria monocytogenes*, which can cause CNS infection, can inserted into the plasma membrane, causing calcium influx and indirectly leading to mitochondrial fission (Stavru et al., 2011). *Helicobacter pylori*, an important pathogen that causes chronic gastritis in various areas of stomach and duodenum, could also cause nervous system inflammation and even promote the occurrence of Alzheimer’s disease (AD). However, the relationship between *Helicobacter pylori* infection and neurological inflammation or AD remains unclear. It has been reported that toxin VacA secreted by *Helicobacter pylori* can migrate across the blood-brain barrier (Suzuki et al., 2019), which could also be inserted in the mitochondrial inner membrane, causing calcium influx and thus indirectly leading to mitochondrial fission (Foo et al., 2010). Since it was proposed that altered balance in mitochondrial fission might be an important mechanism of neuronal dysfunction in the brain tissue of AD patients (Wang et al., 2009; Manczak and Reddy, 2012), it could provide novel insights into how *Helicobacter pylori* targets neural mitochondria and how mitochondrial and neuronal dysfunctions evolve. A recent study also showed that aberrant mitochondria functionality could be a key mediator for the effects of the intestinal microbiota on the progression of depression (Chen and Vitetta, 2020). These studies supported the role that mitochondria plays as an emerging target for bacteria-induced neurological diseases.

**FIGURE 3:**
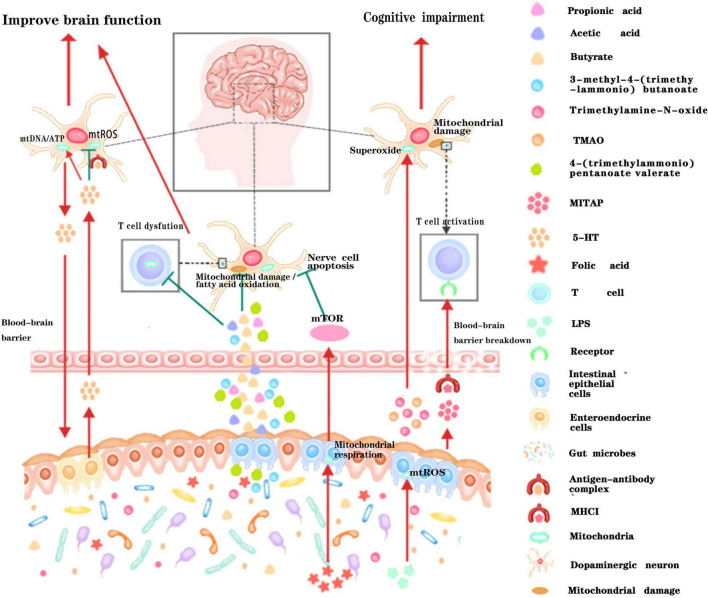
Schematic illustration of the dialog between intestinal microbiota and neural mitochondria across the blood-brain barrier. Metabolites or active small molecules secreted by intestinal microbiota cross the blood-brain barrier and modulate mitochondrial functions through mTOR signaling pathway, ROS signaling pathway, immune pathway, or directly acting on neural mitochondria to influence brain functions. Red arrows indicate enhancement while the green T arrows indicate inhibition.

A large amount of evidence suggest that gut microbiota can also remotely regulate the mitochondrial function of brain tissue through the various metabolites they produced (Figure 3). Short-chain fatty acids (SCFA) generated by gut microbiota can cross the highly selective semipermeable blood-brain barrier (Luu and Visekruna, 2019; Melbye et al., 2019) and directly enter the mitochondria for further oxidative metabolism (Chen and Vitetta, 2020). In addition, supplementation of propionic acid (PA), could defer the progression of Multiple sclerosis (MS) and brain atrophy (Duscha et al., 2020). Since PA can improve mitochondrial function and morphology in competent regulatory T (Treg) cells, and can enter the brain directly, it is rationally speculated that the protective effect of PA on brain tissue may be achieved by improving neural mitochondrial function. Folate produced by gut flora (mainly *Escherichia coli*) could regulate mitochondrial respiration and play an important role in the early development of the nervous system by activating the mammalian target of rapamycin (mTOR) signaling pathway (Silva et al., 2017). Isoallolithocholic acid (IsoalloLCA), distinct derivatives of lithocholic acid, which is also closely related to nervous system diseases, can also induce the production of mitochondrial ROS (Hang et al., 2019). The gut microbiota metabolites 4-(trimethylammonio) pentanoate valerate and 3-methyl-4-(trimethylammonio) butanoate could enter the brain tissue and inhibit the oxidation of mitochondrial fatty acids so as to mediate gut-brain communication (Hulme et al., 2020). Another study found that trimethylamine-N-oxide can also increase mitochondrial damage and superoxide production in mice, thereby accelerating the aging of neurons in the hippocampus of mice, causing and exacerbating aging-related cognitive impairment (Li et al., 2018). Therefore, it is of great theoretical significance to clarify the underlying pathways that gut microbiota metabolites affect brain function by regulating mitochondrial bioactivities, and to reveal how gut microbiota regulate the neuronal functions through dietary metabolism. These studies may provide new drug targets for the ontological enteric treatment of encephalopathy.

## Mitochondria Play an Important Role in Gut Microbiota–Neurotransmitters-Brain Communication

Another strategy of gut microbiota affecting the host’s nervous system is to regulate the host’s neurotransmitter levels, such as gamma-aminobutyric acid (GABA), serotonin (5-HT), and dopamine (DA). These neurotransmitters have been found to be closely in mitochondrial function. For example, GABA can pass through mitochondrial membrane, and regulate citric acid cycle reaction. The distribution mode of GABA is believed to play a critical role in regulating its cytoplasm levels; Conversely, increased mitochondrial activity can reduce GABAergic signaling, resulting in defective social behavior (Kanellopoulos et al., 2020). Recent studies have also shown that 5-HT could promote mitochondrial biogenesis, which is involved in reducing toxic ROS in neurons, protecting buffered neurons from damages caused by cellular stress (Fanibunda et al., 2019). Dopamine has been reported to be associated with mitochondrial dysfunction. Experimental evidence suggests that high dopamine concentrations induce striatal mitochondrial dysfunction through a decrease in mitochondrial respiratory control and loss of membrane potential (Czerniczyniec et al., 2010) and promotes mitochondrial complex I inhibition and leads to neuropsychiatric disorders (Ben-Shachar et al., 2004; Brenner-Lavie et al., 2008). These studies suggested that mitochondria also play an important role in gut microbiota- neurotransmitters-brain communication.

## Mitochondria Play an Important Role in Signal Transmission From Brain to Gut

Reciprocally, mitochondria can also regulate the intestinal microbiota. Studies have shown that mitochondria play an important role in the innate immune response to pathogen infection (Lobet et al., 2015). In addition, mitochondria dysfunction also involves in the regulation of the gut epithelial barrier, allowing transepithelial flux of *Escherichia coli* (Wang et al., 2014). In addition, mitochondrial variants can affect the diversity and composition of intestinal microbiota (Evaldson et al., 1980; Ma et al., 2014; Yardeni et al., 2019). Moreover, mitochondrial chaperone HSP-60 in the neurons regulates anti-bacterial immunity *via* p38 MAP kinase signaling (Jeong et al., 2017). Clinical studies have shown that a large proportion of patients with neurodegenerative diseases, such as Alzheimer’s disease (AD; Haran et al., 2019; Sochocka et al., 2019), Parkinson’s disease (PD; Brudek, 2019; Dumitrescu et al., 2021), and Huntington’s disease (HD; Du et al., 2020; Wasser et al., 2020), often suffer from intestinal inflammation simultaneously. A recent report found that artificial expression of HD-causing protein PolyQ40 in nerve cells of *Caenorhabditis elegans* can induce mitochondrial unfolded protein response in the intestine (Zhang et al., 2018). These results further indicate that mitochondria play an important role in signal transmission between the brain and the gut. Another study found that 5-HT can regulate the colonization of *Turicibacter sanguinis* in the intestine, and *Turicibacter sanguinis* can affect the expression of multiple pathways including lipid and cholesterol metabolism in intestinal (Fung et al., 2019). Combined with the evidence that 5-HT can promote mitochondrial biogenesis and the level of ROS produced by host mitochondria can regulate the diversity of gut flora (Fanibunda et al., 2019), we can deduce that neuron secreted 5-HT regulate the gut flora by regulating the function of intestinal mitochondria. These results suggest that mitochondria mediate two-way communication between gut and brain.

## Conclusion

The understanding of intestinal system has been revolutionized over the past decades, especially in regarding to its physiological and pathological interconnection with brain function. The crosstalk between gut microbiota and central nervous system, which is also known as the microbiota-gut-brain axis have been well elucidated from numerous studies. Numbers of evidence has confirmed that mitochondria can be regulated by the composition of gut microbiota, and mitochondria are also closely related to the physiological and pathological state of the nervous system. In addition, although there are few studies on how gut microbiota directly regulate the physiological and pathological functions of the brain through the mitochondrial pathway, or how the nervous system regulates the composition of gut microbiota through the mitochondrial pathway, the evidence had been gradually reported in recent years. This perspective proposed a hypothetical model about cross-talk between the intestinal microbiome and the neural mitochondria based on the previously known fact that the mitochondria and the bacteria have the evolutionary homology. Symbiont and pathobiont bacteria have the influence to control the neuronal mitochondrial activity. We highlighted the new role of mitochondria in dialog with gut microbiota across the blood-brain barrier, which is one of the important ways of communicating between the brain and gut (Figure 3). The new perspective not only expands our understanding of the brain-gut interaction mechanism, but also provides a new treatment strategy targeting the gut microbiota-mitochondria-brain communication which has the potential to treat a variety of nervous system diseases or digestive system diseases, and may also have a profound impact on future medical treatment.

## Data Availability Statement

The original contributions presented in the study are included in the article/supplementary material, further inquiries can be directed to the corresponding authors.

## Author Contributions

YZ performed the statistical analysis. ZZ, LW, YL, QZ, and YS wrote the manuscript. All authors contributed to the article and approved the submitted version.

## Conflict of Interest

The authors declare that the research was conducted in the absence of any commercial or financial relationships that could be construed as a potential conflict of interest.

## Publisher’s Note

All claims expressed in this article are solely those of the authors and do not necessarily represent those of their affiliated organizations, or those of the publisher, the editors and the reviewers. Any product that may be evaluated in this article, or claim that may be made by its manufacturer, is not guaranteed or endorsed by the publisher.
